# Association between the insulin resistance and all-cause mortality in patients with moderate and severe aortic stenosis: a retrospective cohort study

**DOI:** 10.1186/s12933-023-01975-5

**Published:** 2023-09-02

**Authors:** Rihua Huang, Xinghao Xu, Chaoguang Xu, Shaozhao Zhang, Zhenyu Xiong, Menghui Liu, Yiquan Huang, Han Wen, Yue Guo, Xinxue Liao, Xiaodong Zhuang

**Affiliations:** 1 Department of Cardiology, the First Affiliated Hospital of Sun Yat-Sen University, 58 Zhongshan 2nd Road, Guangzhou, 510080 China; 2 NHC Key Laboratory of Assisted Circulation (Sun Yat-Sen University), Guangzhou, China

**Keywords:** Triglyceride-glucose index, Mortality, Aortic stenosis

## Abstract

**Background:**

The triglyceride-glucose (TyG) index is a reliable surrogate marker of insulin resistance (IR). However, whether the TyG index has prognostic value in patients with moderate to severe aortic stenosis (AS) remains unclear.

**Methods:**

This study enrolled 317 patients with moderate to severe AS at the First Affiliated Hospital of Sun Yat-Sen University. The patients were grouped according to the cut-off value of the TyG index. Cox regression with Firth’s penalized maximum likelihood method and restricted cubic splines regression were conducted to assess the association between the TyG index and all-cause mortality. The added value of the TyG index included in the traditional risk factors model for outcome prediction was also analyzed.

**Results:**

Among 317 patients (mean age 67.70 years, 62.8% male), there was 84 all-cause mortality during a median 38.07 months follow-up. After fully adjusting for confounders, a per-unit increase in the TyG index was associated with a 62% higher all-cause mortality risk (HR 1.622, 95% CI 1.086–2.416, *p* = 0.018). The restricted cubic splines regression model revealed a linear association between the TyG index and the risk of all-cause mortality (*p* for nonlinearity = 0.632). The addition of the TyG index in the basic risk model has an incremental effect on the prediction of mortality [C-statistic change from 0.755 to 0.768; continuous net reclassification improvement (95% CI): 0.299 (0.051–0.546), *p* = 0.017; integrated discrimination improvement: 0.017 (0.001–0.033), *p* = 0.044].

**Conclusions:**

Higher IR assessed by the TyG index was associated with a higher risk of all-cause mortality in patients with moderate and severe AS.

**Supplementary Information:**

The online version contains supplementary material available at 10.1186/s12933-023-01975-5.

## Introduction

Aortic stenosis (AS) is the most common valvular disease worldwide [1]. Over the past decades, the incidence of AS has been rising due to the aging of society [2]. To date, no effective medications can alter the disease progression of AS. Aortic valve replacement (AVR) remains the only guideline-recommended treatment for severe symptomatic AS patients [3, 4]. However, even with advanced interventions, the prognosis after symptom onset is still poor [1]. Therefore, given the socioeconomic impact of AS, early identification of high-risk patients with AS and finding potential therapeutic targets is critically important.

Insulin resistance (IR) is defined as a reduction in tissue sensitivity to normal plasma insulin levels [5], which is a prominent feature of the metabolic syndrome (MetS) and type 2 diabetes mellitus (DM), has been implicated in the pathogenesis of AS [6–8]. Previous evidence indicates that MetS associated with faster AS progression and worse outcomes [7]. Also, DM has been identified as a risk factor for AS and influences AS prognosis [8, 9]. These findings suggest IR may play an important role in AS advancement and prognosis.

Considering traditional IR measurements (such as the hyperinsulinemic-euglycemic clamp technique and homeostasis model assessment for IR) having inherent limitations [10], the triglyceride-glucose (TyG) index has been proposed as a reliable surrogate of IR [11]. Numerous clinical studies have shown that the TyG index is highly associated with increased cardiovascular morbidity [12–15] and mortality [16, 17] in various populations. However, current data are still lacking regarding the associations between the TyG index and all-cause mortality in patients with AS, whether the TyG index has prognostic value in patients with AS remains unclear.

Therefore, the purpose of this study was to evaluate the association between the IR assessed by the TyG index and all-cause mortality in patients with moderate and severe AS.

## Methods

### Study design and participants

The present retrospective study was conducted using patient data from the REal-world Data of CARdiometabolic ProtEcTion study (RED-CARPET study). The RED-CARPET study was a real-world study of hospitalized patients with cardiometabolic diseases at the First Affiliated Hospital of Sun Yat-Sen University, intending to measure associations between established or suspected cardiometabolic disease risk factors and cardiovascular disease (CVD) outcomes and was registered in the Chinese Clinical Trials Registry (registration number: ChiCTR2000039901).

**Fig. 1:**
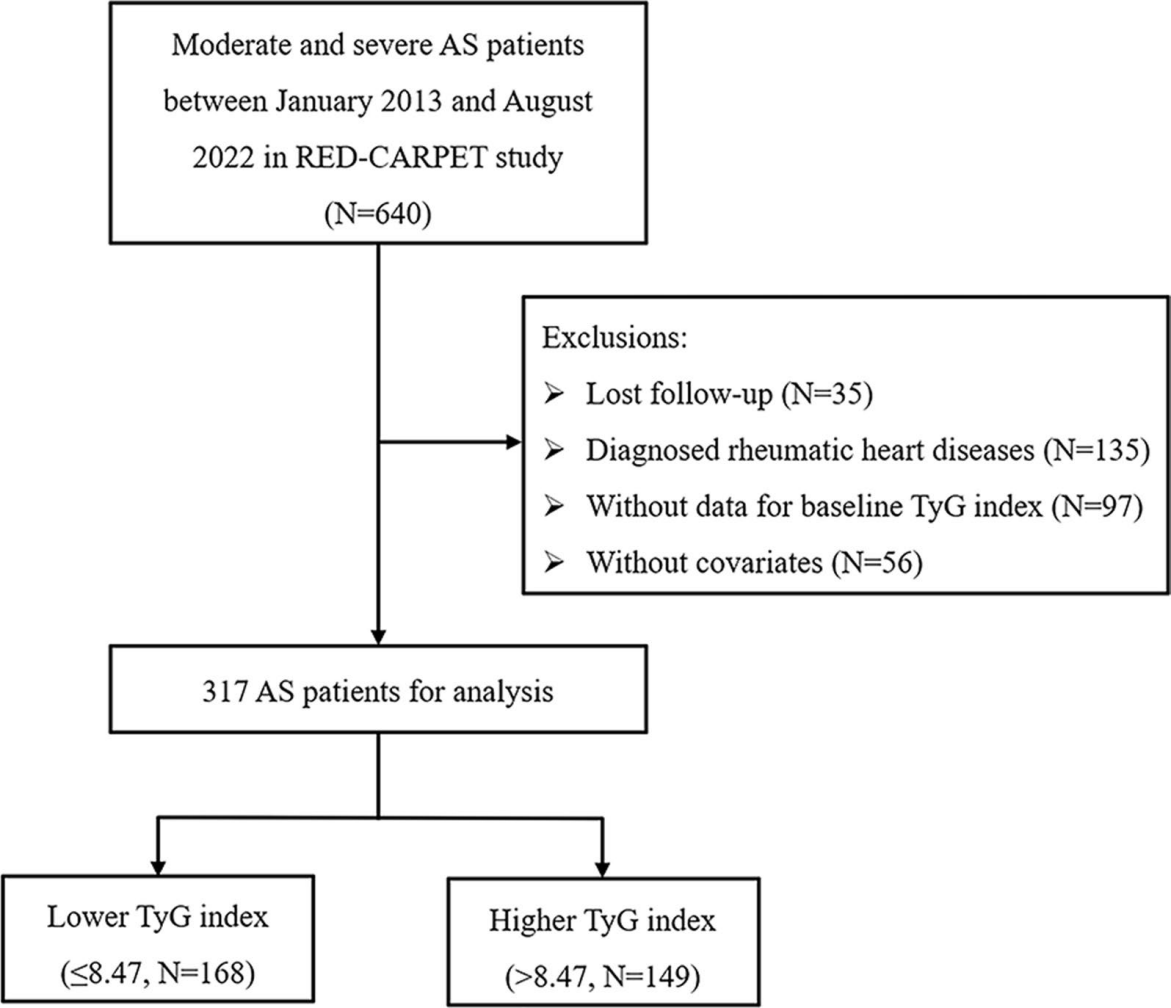
Flow chart for selecting patients with moderate and severe aortic stenosis from RED-CARPET study for analysis

In the present study, a total of 640 patients diagnosed with moderate to severe AS at the First Affiliated Hospital of Sun Yat-Sen University between January 2013 and August 2022 were enrolled retrospectively. The inclusion criteria were as follows: (1) over 18 years old; (2) diagnosis of moderate to severe AS by echocardiography based on guidelines [3]. Moderate to severe AS was defined as peak aortic jet velocity (Vmax) > 3 m/s, mean aortic pressure gradient (MG) > 20 mm Hg, or aortic valve area (AVA) ≤ 1.5 cm^2^. (3) had no history of aortic valve replacement at baseline. The exclusion criteria were as follows: (1) without data for baseline TyG index (N = 97); (2) diagnosis of rheumatic heart diseases (N = 135); (3) diagnosis of malignant tumor (N = 0); (4) without covariates (N = 56); (5) lost follow-up (N = 35). Finally, 317 patients with moderate to severe AS were included for analysis (Fig. 1). This study was conducted following the Declaration of Helsinki and was approved by the Ethics Review Committee of the First Affiliated Hospital of Sun Yat-Sen University. The clinical data for this study were gathered through electronic medical records, and the follow-up was done via phone, with verbal informed consent approved by the institutional ethics committee.

### Data collection and definitions

Clinical data, including age, gender, height, weight, history of smoking and alcohol, and current medication use were obtained through self-reporting by patients at the time of admission. History of hypertension, diabetes, coronary heart disease (CHD), and stroke were also self-reported, and further validated by medical professionals based on hospitalization records such as blood tests or imaging examinations. The information about subsequent AVR surgery was obtained through electronic medical records or telephone interviews. The systolic blood pressure (SBP) and diastolic blood pressure (DBP) were measured by the medical staff with a sphygmomanometer after the patient rested for 5 minutes in the morning. The body mass index (BMI) was computed by dividing the weight (kg) by height squared (m^2^). Laboratory parameters including total cholesterol (TC), high-density lipoprotein cholesterol (HDL-C), low-density lipoprotein cholesterol (LDL-C), triglyceride (TG), and fasting plasma glucose (FBG), were analyzed by standard techniques using venous blood samples obtained after overnight fasting (> 8 h). Hypertension was defined as SBP ≥ 140 mm Hg and/or DBP ≥ 90 mm Hg, or using of antihypertensive medication and self-reported history of hypertension. Diabetes mellitus was defined as FPG ≥ 7.0 mmol/L, or HbA1c ≥ 6.5%, and self-reported history of type 2 diabetes mellitus (T2DM). The TyG index was calculated as ln(TG [mg/dl] × FBG [mg/dl]/2).

### Echocardiography

Comprehensive transthoracic echocardiography was conducted with commercially available ultrasound equipment following the American Society of Echocardiography guidelines [18]. Vmax and MG were calculated using the modified Bernoulli equation. AVA was computed by the continuity equation. The severity of AS was defined based on the following criteria [3, 4]: (1) moderate AS: AVA > 1 cm^2^ and ≤ 1.5 cm^2^, or Vmax > 3 m/s and ≤ 3.9 m/s, or MG > 20 mm Hg and ≤ 39 mm Hg; (2) severe AS: AVA ≤ 1 cm^2^, or Vmax ≥ 4 m/s, or MG ≥ 40 mm Hg. Pulmonary artery pressure (PAP) was also calculated using the Bernoulli equation to measure the pressure gradient between the right ventricle and the right atrium. Pulmonary arterial hypertension (PAH) was defined as a pulmonary artery pressure ≥ 25 mm Hg. The left ventricular (LV) chamber size, as well as systolic and diastolic function, was also assessed in line with the latest guidelines [19, 20].

### Outcome

The primary endpoint of this study was all-cause mortality, collected by trained medical personnel contacting patients or their family members by phone. Follow-up time was calculated as the period between the date of AS diagnosis and the date of death or last follow-up. The follow-up of all patients who were included in this study was completed in December 2022.

### Statistical analysis

Continuous variables are presented as mean ± standard deviation (SD) or median with interquartile ranges based on normal or non-normal distribution. Categorical data were presented as numbers and percentages. Patients were grouped according to the optimal TyG index in predicting the primary endpoint in the total study sample. The Student’s t-test or Mann–Whitney U test was used to evaluate group differences for continuous variables, and the Pearson chi-square test or Fisher’s exact test was used for categorical variables appropriately. The associations between the TyG index and cardiovascular risk factors were evaluated using the Pearson correlation test or Spearman’s rank correlation test. The Pearson correlation test is used to evaluate the relationship between two normally distributed continuous variables, whereas the Spearman rank correlation test is used when non-normally distributed continuous or categorical data are being studied. The optimal cutoff point value of the TyG index for predicting the primary endpoint was determined using receiver-operating characteristic (ROC) curve analysis. The Kaplan-Meier method was used to calculate the cumulative incidence of all-cause mortality according to the optimal cutoff point of the TyG index, and differences between groups were assessed using the log-rank test. Cox regression with Firth’s penalized maximum likelihood method was conducted to evaluate hazard ratios (HR) and 95% confidence interval (CI) for the association between the TyG index with all-cause mortality. Three multivariable models were built and used to adjust for potential confounders of all-cause mortality. Model 1 was adjusted for sex and age at baseline. Model 2 was additionally adjusted for BMI, LDL-C, smoking status, drinking status, SBP, DBP, AS severity, PAH, LV ejection fraction, bicuspid aortic valve, and AVR. Model 3 was further adjusted for variables in model 2 plus diabetes mellitus, CHD, antiplatelets and statins use. A restricted cubic spline regression model with three knots was used to assess the nonlinear dose-response relationship between the TyG index and incident all-cause mortality. Further subgroup analyses stratified by baseline sex, age (≤ 70 and > 70 years), BMI (< 24 and ≥ 24 kg/m^2^), diabetes mellitus, hypertension, CHD, AS severity (moderate and severe), AVR, and use of hypoglycemic medications were employed to examine the consistency of the prognostic impact of TyG index for all-cause mortality. Given the noticeable impact of hypoglycemic medications on our analysis, further sensitivity analyses incorporated additional adjustments for the use of insulin and oral hypoglycemic agents. The incremental prognostic value of the TyG index was investigated using Harrell C statistic, net reclassification statistics, and integrated discrimination improvement (IDI) in fitted Cox regression models.

All analyses were conducted in R version 4.1.3 (R Foundation for Statistical Computing, Vienna, Austria) and Stata 17.0 (Stata Corp LLC, Texas, USA). A two-sided P value < 0.05 was considered statistically significant.

## Results

### Baseline characteristics

The ROC curve analysis showed that the optimal cutoff point of the TyG index for predicting primary outcome was 8.47 (Additional file 1: Table S1). Table 1 shows the baseline characteristics of 317 moderate and severe AS patients grouped by the optimal cutoff point of the TyG index. The mean age of the total patients was 67.70 ± 12.31 years and 199 (62.8%) were male. Patients with TyG index ≥ 8.47 had higher levels of TC, LDL-C, TG, FPG, DBP, and BMI, a lower level of HDL-C, and a higher proportion of drinker, diabetes mellitus, oral hypoglycemic agents and insulin use, compared to patients with TyG index < 8.47. The echocardiographic findings of the moderate and severe AS patients are shown in Additional file 1: Table S2. Patients with higher TyG index appeared to have higher relative wall thickness and higher prevalence of pulmonary arterial hypertension, but no significant differences were found in other echocardiographic characteristics. Relative to the excluded patients of the overall cohort, the included patients were more likely to be older, male, and have hypertension, diabetes mellitus, and CHD (Additional file 1: Table S3).

**Table 1:** Baseline clinical characteristics of patients stratified by the optimal cutoff point of the TyG index

	Total population (n = 317)	Lower TyG index (≤ 8.47, n = 168)	Higher TyG index (> 8.47, n = 149)	*p* value
TyG index	8.52 ± 0.62	8.06 ± 0.30	9.03 ± 0.47	< 0.001
Age	67.70 ± 12.31	67.97 ± 12.92	67.40 ± 11.62	0.683
Sex				
Female	118 (37.2)	63 (37.5)	55 (36.9)	1.000
Male	199 (62.8)	105 (62.5)	94 (63.1)	
FPG, mmol/L	5.71 ± 2.49	4.87 ± 1.03	6.65 ± 3.21	< 0.001
Total Cholesterol, mmol/L	4.51 ± 1.21	4.27 ± 1.0	4.79 ± 1.30	< 0.001
TG, mmol/L	1.34 ± 0.83	0.87 ± 0.23	1.86 ± 0.94	< 0.001
LDL-C, mmol/L	2.83 ± 0.87	2.63 ± 0.76	3.05 ± 0.92	< 0.001
HDL-C, mmol/L	1.14 ± 0.34	1.20 ± 0.34	1.07 ± 0.32	0.001
SBP, mmHg	132.11 ± 21.59	130.62 ± 21.15	133.80 ± 22.03	0.191
DBP, mmHg	72.38 ± 12.95	70.30 ± 12.54	74.73 ± 13.04	0.002
BMI, kg/m^2^	23.23 ± 3.66	22.29 ± 3.47	24.28 ± 3.59	< 0.001
Smoking	96 (30.3)	48 (28.6)	48 (32.2)	0.560
Drinking	59 (18.6)	24 (14.3)	35 (23.5)	0.050
Hypertension	171 (53.9)	86 (51.2)	85 (57.0)	0.352
Diabetes mellitus	64 (20.2)	21 (12.5)	43 (28.9)	< 0.001
CHD	108 (34.1)	49 (29.2)	59 (39.6)	0.066
Stroke	29 (9.1)	16 (9.5)	13 (8.7)	0.959
Statins	151 (47.6)	76 (45.2)	75 (50.3)	0.427
Hypoglycemic medications				
Oral hypoglycemic agents	48 (15.1)	14 (8.3)	34 (22.8)	0.001
Insulin	36 (11.4)	10 (6.0)	26 (17.4)	0.002
Antihypertension medication	230 (72.6)	118 (70.2)	112 (75.2)	0.392
Antiplatelets	142 (44.8)	71 (42.3)	71 (47.7)	0.395
Aortic valve replacement	144 (45.4)	75 (44.6)	69 (46.3)	0.854
Aortic stenosis severity				
Moderate	94 (29.7)	50 (29.8)	44 (29.5)	1.000
Severe	223 (70.3)	118 (70.2)	105 (70.5)	

Data are shown as mean ± SD or n (%). Baseline characteristics of the 317 eligible patients from the RED-CARPET study, stratified by the optimal cutoff point of triglyceride -glucose index. TyG, triglyceride-glucose; BMI, body mass index; SBP, systolic blood pressure; DBP, diastolic blood pressure; FPG, fasting plasma glucose; HDL-C, high-density lipoprotein cholesterol; LDL-C, low-density lipoprotein cholesterol; TG, triglyceride; CHD, coronary heart disease

### Correlations between the TyG index and traditional cardiovascular risk factors

Table 2 shows the correlation between the TyG index and traditional risk factors for CVD. Among the moderate and severe AS patients, the TyG index was found to be positively correlated with BMI, FBG, TG, TC, LDL-C, and DBP, but negatively correlated with HDL-C. There was no significant correlation between the TyG index and age, gender, or SBP.

**Table 2:** Correlations between the TyG index and traditional cardiovascular risk factors

Variable	Correlation coefficient	*p* Value
Age	− 0.027	0.631
Sex, male	− 0.058	0.299
BMI	0.297	< 0.001
FBG	0.575	< 0.001
TG	0.797	< 0.001
TC	0.246	< 0.001
LDL-C	0.253	< 0.001
HDL-C	− 0.208	< 0.001
SBP	0.046	0.409
DBP	0.217	< 0.001

BMI, body mass index; FPG, fasting plasma glucose; TG, triglyceride; TC, total cholesterol; HDL-C,  high-density lipoprotein cholesterol; LDL-C, low-density lipoprotein cholesterol; SBP, systolic blood pressure; DBP, diastolic blood pressure

### Association between the TyG index and all-cause mortality

During a median follow-up of 38.07 months, 84 all-cause mortality were documented for analysis. Table 3 shows the Cox regression with Firth’s penalized maximum likelihood analysis of the association between the TyG index and all-cause mortality. Notably, although the association between the TyG index and all-cause mortality was non-significant in model 1 and Kaplan-Meier analysis, highly significant values were found after further adjusting for potential confounders in models 2 and 3 (Table 3, Additional file 1: Fig. S1). In the fully adjusted model 3, per 1-unit higher TyG index was associated with a 62% higher risk of all-cause mortality (HR 1.622, 95% CI 1.086–2.416, *p* = 0.018). Also, compared to the lower TyG index group, the higher TyG index group was associated with a 1.746 times higher risk of all-cause mortality (HR 1.746, 95% CI 1.093–2.798, *p* = 0.019). Additionally, the association between the TyG index and all-cause mortality remains robust after further adjustments for the use of insulin and oral hypoglycemic agents (Additional file 1: Table S4)

**Table 3:** Association between the TyG index and all-cause mortality in the moderate to severe AS patients*

	Events/Total	Model 1		Model 2		Model 3	
		HR (95%CI)	*p* value	HR (95%CI)	*p* value	HR (95%CI)	*p* value
Continuous (per unit)	84/317 (26.5)	1.279 (0.887, 1.821)	0.184	1.654 (1.119, 2.431)	0.012	1.622 (1.086, 2.416)	0.018
Lower TyG index	37/168 (22.0)	Reference	–	Reference	–	Reference	–
Higher TyG index	47/149 (31.5)	1.493 (0.970, 2.311)	0.067	1.802 (1.136, 2.867)	0.012	1.746 (1.093, 2.798)	0.019

*Cox regressions with Firth’s penalized maximum likelihood method were used

Model 1 Adjusted by sex, age

Model 2 Adjusted by model 1 + body mass index, low-density lipoprotein cholesterol, systolic blood pressure, diastolic blood pressure, smoking status, drinking status, aortic stenosis severity, pulmonary arterial hypertension, LV ejection fraction, bicuspid aortic valve, and aortic valve replacement

Model 3 Adjusted by model 2 + diabetes mellitus, CHD, antiplatelets, and statins use

TyG, triglyceride-glucose; HR, hazard ratio; CI, confidence interval

**Fig. 2:**
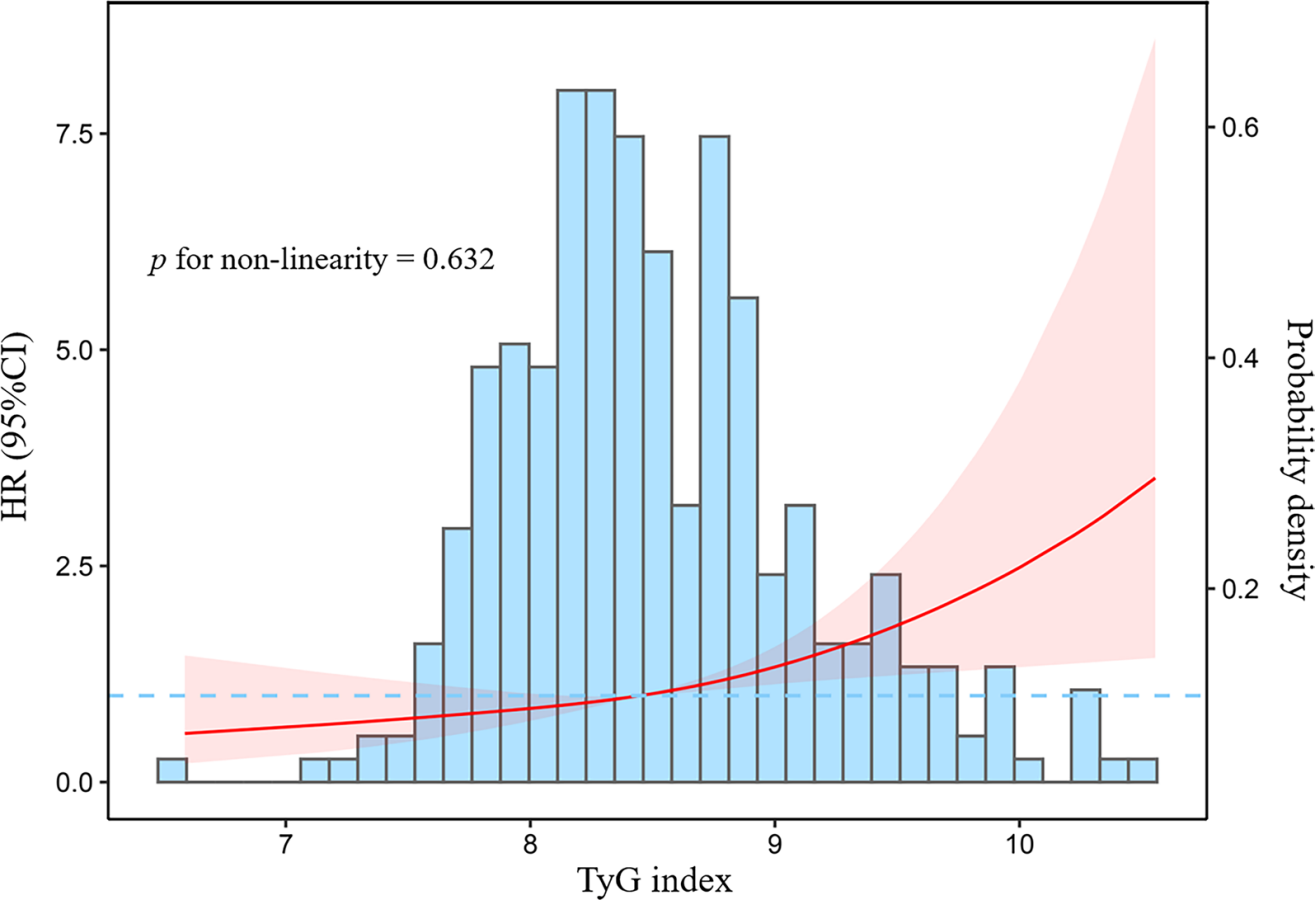
Adjusted hazard ratios for incident all-cause mortality by baseline TyG index. The blue columns present the distribution density of the TyG index. The blue dashed line represents HR = 1. The red solid line shows the HR value. The red shaded area represents the 95% CI. The hazard ratio was computed with a median baseline TyG index level of 8.47. HR, hazard ratios; CI, confidence interval

**Fig. 3:**
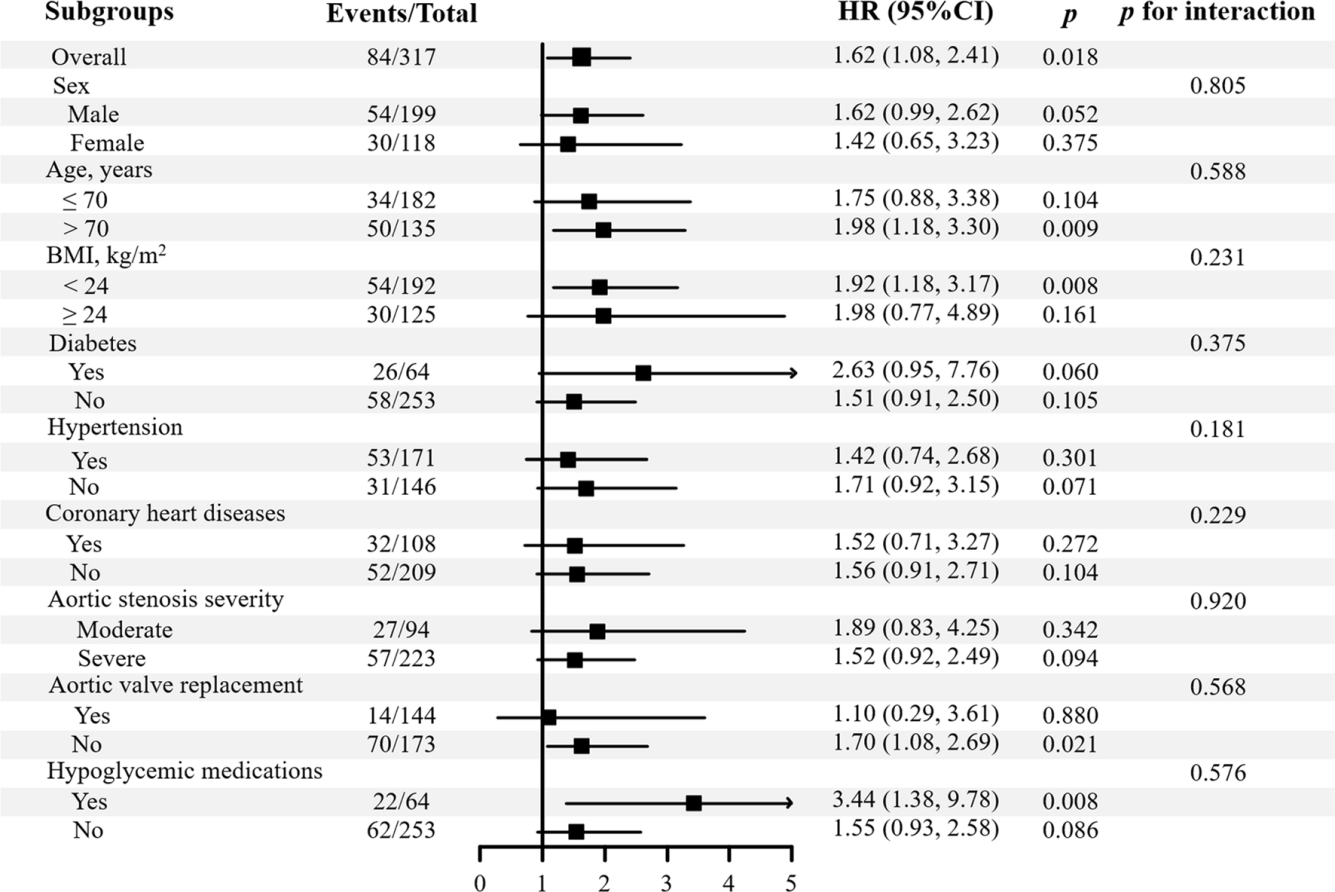
Subgroup analysis of the association between baseline TyG index and incident all-cause mortality. HR, hazard ratios; CI, confidence interval; BMI, body mass index

Multivariable adjusted restricted cubic splines regression models also showed a linear association between the TyG index with the risk of all-cause mortality (*p* for non-linearity = 0.632) (Fig. 2). Subgroup analyses were conducted in sex (male or female), age (≤ 70 or > 70 years), BMI (< 24 or ≥ 24 kg/m^2^), diabetes mellitus (yes or no), hypertension (yes or no), CHD (yes or no), AS severity (moderate or severe), AVR (yes or no) and use of hypoglycemic medications (yes or no) (Fig. 3). The positive effect of the TyG index on all-cause mortality was consistent after adjusting for the confounders across all subgroups. There were no significant interactions in the subgroups (all *p* for interaction > 0.05).

### Incremental predictive value of TyG index

The predictive value of the TyG index for all-cause mortality was assessed using fitted multivariable Cox regression model components (Table 4). In the total study sample, adding the TyG index to the fitted risk model targeting all-cause mortality yielded a significant improvement with NRI of 0.299 (*p* = 0.017) and IDI of 0.017 (*p* = 0.044). In addition, the C-index of the basic risk model [0.755 (0.700–0.810), *p* < 0.001] changed after the addition of the TyG index [0.768 (0.713–0.823), *p* < 0.001].

**Table 4:** Performance of the model with TyG index to predict all-cause mortality in moderate to severe AS patients

	C-index		Continuous-free NRI		IDI	
	Index	*p* value	Index	*p* value	Index	*p* value
Basic model	0.755 (0.700–0.810)	< 0.001	Ref.		Ref.	
Basic model + TyG index	0.768 (0.713–0.823)	< 0.001	0.299 (0.051–0.546)	0.017	0.017 (0.001–0.033)	0.044

TyG, triglyceride-glucose; NRI, net reclassification improvement; IDI, integrated discrimination improvement;

The basic model included: sex, age, body mass index, low-density lipoprotein cholesterol, systolic blood pressure, diastolic blood pressure, smoking status, drinking status, aortic stenosis severity, pulmonary arterial hypertension, left ventricular ejection fraction, bicuspid aortic valve, aortic valve replacement, diabetes mellitus, CHD, antiplatelets, and statins use

## Discussion

To our knowledge, this is the first study to evaluate the relationship between IR assessed by the TyG index and prognosis in patients with moderate and severe AS. The main finding showed that a higher TyG index was linearly and significantly associated with a higher risk of all-cause mortality in these patients. In addition, adding the TyG index to a traditional risk factors model provided incremental predictive capacity for all-cause mortality.

IR is the core pathogenic feature of MetS, a cluster of disorders including impaired glucose metabolism, dyslipidemia, obesity, and hypertension, all of which lead to increased CVD and mortality [21, 22]. The hyperinsulinemic-euglycemic clamp is the gold standard test for assessing IR; however, its application is limited owing to its complexity and cost [10]. Recently, the TyG index, calculated simply using fasting blood glucose and triglycerides, has been widely regarded as a simple and reliable marker of IR [11]. Growing evidence shows that the TyG index is associated with all-cause and cardiovascular mortality among the general population [23, 24] and patients with different cardiometabolic disorders [16, 25–27]. For example, in a recent study that included a total of 1618 patients from the Medical Information Mart for Intensive Care III, they found that the TyG index was significantly linearly associated with the risk of all-cause mortality in critically ill patients with CHD [16]. Another study also found a significant association between the TyG index and long-term all-cause mortality and major adverse cardiac events in elderly acute coronary syndrome patients [28]. Moreover, one prospective analysis of the Coronary Artery Risk Development in Young Adults (CARDIA) study reported that every unit increase in TyG index was associated with an 85% higher all-cause mortality risk (HR 1.85, 95% CI 1.45–2.36) in the young healthy adults [23]. However, the prognostic impact of the IR evaluated by the TyG index among patients with AS has not been evaluated. Several studies suggested that IR, a central feature of MetS and diabetes mellitus, may play an important role in the progression of AS and as a risk factor for adverse outcomes in AS patients. A retrospective study, including 105 consecutive patients with at least moderate AS, found that MetS is a strong independent predictor of event-free survival with an odds ratio of 3.85 (95% CI 1.96–7.58) compared with those without MetS [7]. Another recent study, including 2703 patients with mild to severe AS from France, showed that DM was strongly associated with death from heart failure and sudden death in patients with severe AS [29]. Nevertheless, current studies are still lacking to directly evaluate the relationship between IR and mortality in patients with AS.

In the present study, we first demonstrated that a higher IR assessed by the TyG index was significantly associated with a higher risk of all-cause mortality after fully adjusting potential confounders in patients with moderate and severe AS. Multivariable adjusted restricted cubic splines regression models further showed a linear relationship between the TyG index with the risk of all-cause mortality. Subgroup analysis also presented consistent results. Moreover, the addition of the TyG index to the fitted risk model including traditional risk factors provided incremental predictive capacity. Of note, the association between the TyG index and all-cause mortality failed to reach statistical significance in the adjusted model 1 and the Kaplan-Meier curves. This could be because of the short follow-up period, the small sample size, or the inherent selection bias of using hospital data. Further studies with larger sample sizes and extended follow-ups are required to provide more robust evidence to support our findings. Overall, a highly significant association between the TyG index and all-cause mortality was found in patients with moderate and severe AS.

Although the exact biological mechanisms explaining the relationship between the TyG index and mortality remain unclear, several possible mechanisms have been proposed. Firstly, the TyG index is highly correlated with the gold standard method for IR assessment [11] and is considered a simple and reliable marker of IR, which can lead to metabolic disorders such as hyperglycemia and dyslipidemia. These metabolic alterations have been reported by epidemiological and genetic studies to be the causes of CVD and all-cause mortality [30]. In addition, the TyG index has been proven to be closely related to the other traditional risk factors for CVD [31], and the present study also found consistent results. Therefore, individuals with higher TyG index levels may be more likely to develop cardiometabolic morbidity and consequently significantly lower life expectancy [32]. Furthermore, IR has been found to cause endothelial dysfunction, affect cardio-metabolism, enhance foam cell formation, trigger oxidative stress, and exacerbate inflammatory responses, which play important roles in the development of CVD [5, 33, 34]. Regardless, further studies are still needed to elucidate the exact mechanisms of the association between the TyG index and mortality.

The results of our study have important clinical implications. First, we have found a significant linear relationship between the TyG index and future mortality in moderate and severe AS patients, and adding the TyG index to the traditional risk factors model has an incremental effect on the prediction of mortality. These results highlight that the TyG index may serve as an effective tool for risk stratification and management in this high-risk patient population. Second, higher TyG levels are associated with a higher prevalence of CVD risk factors, indicating that patients with higher TyG levels should take a comprehensive approach to risk management. In addition, several cardiovascular outcome trials have shown that IR-lowering therapies are a promising intervention for patients at risk of adverse cardiovascular events [35–37]. For example, pioglitazone, a potent insulin sensitizer, has been proven to reduce cardiovascular complications in patients without diabetes who had IR along with a recent history of ischemic stroke or transient ischemic attack [36]. Glucagon-like peptide 1 analogs have also been shown to reduce the risk of major adverse cardiac events, in part due to their direct effects on changes in vascular redox status and IR [37]. Therefore, the treatment of IR may help to improve the clinical prognosis. Additional prospective studies, of course, are needed to determine whether more aggressive IR treatment can reduce the prognosis of AS patients.

This study also has several limitations as follows. First, this was a single-center retrospective study, therefore causality could not be definitively established and potential bias could have been introduced. Second, the sample size was relatively small and the incidence of all-cause mortality was relatively low, which may have led to an underpowered analysis. Third, the insulin levels and the hyperinsulinemic-euglycemic clamp test were not measured in this study, so we could not compare the TyG index with the homeostasis model assessment for IR (HOMA-IR) and the hyperinsulinaemic-–euglycaemic clamp test. Fourth, laboratory parameters were only detected once at admission, which may have resulted in a bias due to measurement error. Fifth, although multivariable has been adjusted in the Cox regression model, residual confounders were still possible. Sixth, the included patients were relatively unhealthy compared to the excluded patients, which could lead to a potential selection bias. Finally, the study was based on Chinese patients and needs further validation in other ethnic studies.

## Conclusion

In summary, this is the first study to report that a higher TyG index was associated with a high risk of all-cause mortality in patients with moderate to severe AS. Our findings suggest that the TyG index could be used as a predictor of all-cause mortality and therapies aimed at improving insulin resistance may help to improve the clinical prognosis in the moderate to severe AS patients.

### Supplementary Information

**Additional file 1: Figure S1.** Cumulative incidence of all-cause mortality according to the optimal cutoff point of the TyG index. **Table S1.** The ROC curve analysis determined the optimal cutoff value of the TyG index for predicting all-cause mortality in patients with aortic stenosis. **Table S2.** Echocardiographic characteristics of patients according to the optimal cutoff point of TyG index. **Table S3.** Baseline characteristics of excluded and included patients. **Table S4.** Association between the TyG index and all-cause mortality in the moderate to severe AS patients with further adjustment for use of insulin and oral hypoglycemic agents.

## Data Availability

The datasets used and/or analyzed during the current study are available from the corresponding author upon reasonable request.

## Abbreviations

RED-CARPET study: REal-world Data of CARdiometabolic ProtEcTion study
IR: Insulin resistance
TyG: Triglyceride-glucose
AS: Aortic stenosis
CVD: Cardiovascular disease
CHD: Coronary heart disease
MetS: Metabolic syndrome
SBP: Systolic blood pressure
DBP: Diastolic blood pressure
BMI: Body mass index
TC: Total cholesterol
TG: Triglyceride
LDL-C: Low-density lipoprotein cholesterol
HDL-C: High-density lipoprotein cholesterol
FBG: Fasting blood glucose
PAH: Pulmonary arterial hypertension
LV: Left ventricular
AVR: Aortic valve replacement
