# Supplementary material for: Association between the insulin resistance and all-cause mortality in patients with moderate and severe aortic stenosis: a retrospective cohort study
Source: Cardiovasc Diabetol. 2023 Sep 2;22:238. doi: 10.1186/s12933-023-01975-5 (PMC10475183; doi:10.1186/s12933-023-01975-5)
Supplement: Supplementary file 1 — Additional file 1: Figure S1. Cumulative incidence of all-cause mortality according to the optimal cutoff point of the TyG index. Table S1. The ROC curve analysis determined the optimal cutoff value of the TyG index for predicting all-cause mortality in patients with aortic stenosis. Table S2. Echocardiographic characteristics of patients according to the optimal cutoff point of TyG index. Table S3. Baseline characteristics of excluded and included patients. Table S4. Association between the TyG index and all-cause mortality in the moderate to severe AS patients with further adjustment for use of insulin and oral hypoglycemic agents. [file 12933_2023_1975_MOESM1_ESM.docx]

**Association between the insulin resistance and all-cause mortality in patients with moderate and severe aortic stenosis: a retrospective cohort study**

Rihua Huang^1,2†^, Xinghao Xu^1,2†^, Chaoguang Xu^1,2†^, Shaozhao Zhang^1,2^, Zhenyu Xiong^1,2^, Menghui Liu^1,2^, Yiquan Huang^1,2^, Han Wen^1,2^, Yue Guo^1,2^, Xinxue Liao^1,2*^ and Xiaodong Zhuang^1,2*^

^1^ Department of Cardiology, the First Affiliated Hospital of Sun Yat-Sen University.

^2^ NHC Key Laboratory of Assisted Circulation (Sun Yat-Sen University).

*Additional file 1: Figure S1.* Cumulative incidence of all-cause mortality according to the optimal cutoff point of the TyG index.

*Additional file 1: Table S1*. The ROC curve analysis determined the optimal cutoff value of the TyG index for predicting all-cause mortality in patients with aortic stenosis.

*Additional file 1: Table S2*. Echocardiographic characteristics of patients according to the optimal cutoff point of TyG index.

*Additional file 1: Table S3*. Baseline characteristics of excluded and included patients.

*Additional file 1: Table S4.* Association between the TyG index and all-cause mortality in the moderate to severe AS patients with further adjustment for use of insulin and oral hypoglycemic agents.

*Additional file 1: Figure S1.* Cumulative incidence of all-cause mortality according to the optimal cutoff point of the TyG index.


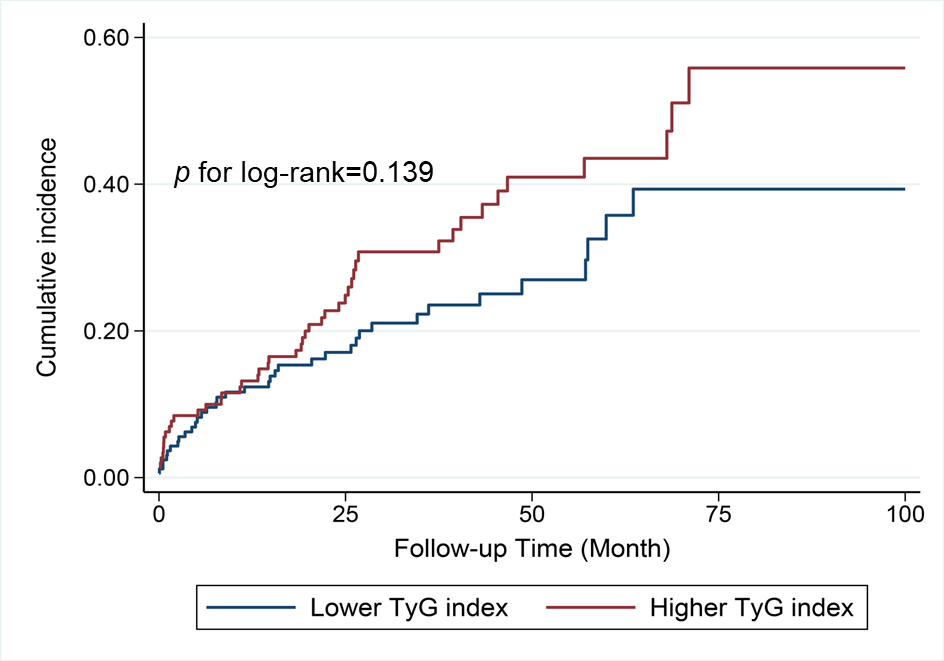


*Additional file 1: Table S1*. The ROC curve analysis determined the optimal cutoff value of the TyG index for predicting all-cause mortality in patients with aortic stenosis.

| Test | optimal cut-off value | Sensitivity | Specificity | AUC |
| --- | --- | --- | --- | --- |
| TyG index | 8.47 | 0.562 | 0.560 | 0.53 |

ROC curve, receiver operating characteristic curve; AUC, area under the curve; TyG index, triglyceride-glucose index.

*Additional file 1: Table S2.* Echocardiographic characteristics of patients according to the optimal cutoff point of TyG index.

|  | **Total population**  **(n=317)** | **Lower TyG index**  **(≤8.47, n=168)** | **Higher TyG index**  **(>8.47, n=149)** | ***p* value** |
| --- | --- | --- | --- | --- |
| AS severity |  |  |  | 1 |
| moderate AS | 94 (29.7) | 50 (29.8) | 44 (29.5) |  |
| severe AS | 223 (70.3) | 118 (70.2) | 105 (70.5) |  |
| Aortic regurgitation | 290 (91.5) | 154 (91.7) | 136 (91.3) | 1 |
| Mitral stenosis | 30 (9.5) | 14 (8.3) | 16 (10.7) | 0.591 |
| Mitral regurgitation | 238 (75.1) | 132 (78.6) | 106 (71.1) | 0.163 |
| Bicuspid valve | 60 (18.9) | 30 (17.9) | 30 (20.1) | 0.709 |
| Pulmonary arterial hypertension | 108 (34.1) | 70 (41.7) | 38 (25.5) | 0.017 |
| Peak aortic jet velocity, m/s | 4.10 ± 0.87 | 4.09 ± 0.91 | 4.10 ± 0.82 | 0.925 |
| Peak aortic gradient, mmHg | 69.74 ± 29.99 | 70.15 ± 32.07 | 69.27 ± 27.55 | 0.794 |
| Mean aortic gradient, mmHg | 42.21 ± 19.09 | 42.59 ± 20.57 | 41.79 ± 17.35 | 0.711 |
| Aortic valve area, cm^2^ | 0.95 ± 0.32 | 0.96 ± 0.34 | 0.93 ± 0.29 | 0.366 |
| LVEDD, mm | 52.12 ± 9.27 | 53.02 ± 9.96 | 51.11 ± 8.34 | 0.068 |
| LVESD, mm | 33.78 ± 9.45 | 34.76 ± 10.30 | 32.67 ± 8.28 | 0.051 |
| LV ejection fraction, % | 63.95 ± 12.60 | 63.19 ± 13.62 | 64.81 ± 11.33 | 0.254 |
| Left atrial diameter, mm | 40.13 ± 7.51 | 40.37 ± 8.36 | 39.86 ± 6.45 | 0.547 |
| IVS, mm | 13.27 ± 3.09 | 12.97 ± 3.41 | 13.61 ± 2.65 | 0.065 |
| LVPWT, mm | 11.04 ± 2.91 | 10.82 ± 2.15 | 11.29 ± 3.57 | 0.149 |
| Stroke volume, mL | 81.94 ± 31.97 | 84.60 ± 36.30 | 78.57 ± 25.23 | 0.174 |
| Left ventricular mass, g | 263.95 ± 114.05 | 265.15 ± 119.83 | 262.60 ± 107.54 | 0.843 |
| LVMI, g/m^2^ | 162.91 ± 67.32 | 166.70 ± 69.41 | 159.14 ± 65.17 | 0.318 |
| Relative wall thickness, cm | 0.44 ± 0.14 | 0.42 ± 0.11 | 0.45 ± 0.17 | 0.033 |
| Mean E/e’ | 16.35 ± 7.89 | 17.05 ± 8.77 | 15.43 ± 6.48 | 0.136 |

Data are shown as mean ± SD or n (%). Baseline characteristics of the 317 eligible patients from the RED-CARPET study, stratified by the optimal cutoff point of triglyceride glucose index. AS = aortic stenosis, LVEDD = Left ventricular end-diastolic diameter, LVESD = Left ventricular end-systolic diameter, LVPWT = Left ventricular posterior wall thickness, IVS = Interventricular septum thickness, LVMI = Left ventricular mass index.

*Additional file 1: Table S3.* Baseline characteristics of excluded and included patients.

|  | **Excluded (n = 323)** | **Included (n = 317)** | ***p* value** |
| --- | --- | --- | --- |
| Age | 60.68 ± 14.38 | 67.70 ± 12.31 | <0.001 |
| Sex |  |  |  |
| Female | 157 (49.5) | 118 (37.2) | 0.002 |
| Male | 160 (50.5) | 199 (62.8) |  |
| FPG, mmol/L | 5.53 ± 3.00 | 5.71 ± 2.49 | 0.423 |
| Total Cholesterol, mmol/L | 4.40 ± 1.39 | 4.51 ± 1.21 | 0.354 |
| TG, mmol/L | 1.17 ± 0.60 | 1.34 ± 0.83 | 0.018 |
| LDL-C, mmol/L | 2.84 ± 0.99 | 2.83 ± 0.87 | 0.898 |
| HDL-C, mmol/L | 1.08 ± 0.37 | 1.14 ± 0.34 | 0.071 |
| TyG index | 8.39 ± 0.62 | 8.52 ± 0.62 | 0.022 |
| SBP, mmHg | 123.01 ± 59.82 | 132.11 ± 21.59 | 0.011 |
| DBP, mmHg | 68.79 ± 11.72 | 72.38 ± 12.95 | <0.001 |
| BMI, kg/m^2^ | 22.87 ± 3.64 | 23.23 ± 3.66 | 0.259 |
| Smoking | 76 (24.4) | 96 (30.3) | 0.120 |
| Drinking | 46 (14.7) | 59 (18.6) | 0.233 |
| Hypertension | 101 (32.4) | 171 (53.9) | <0.001 |
| Diabetes mellitus | 41 (13.1) | 64 (20.2) | 0.023 |
| CHD | 45 (14.4) | 108 (34.1) | <0.001 |
| Stroke | 22 (7.1) | 29 (9.1) | 0.421 |
| Statins | 64 (20.6) | 151 (47.6) | <0.001 |
| Hypoglycemic medications |  |  |  |
| Oral hypoglycemic agents | 25 (8.1) | 48 (15.1) | 0.009 |
| Insulin | 23 (7.4) | 36 (11.4) | 0.121 |
| Antihypertension medication | 170 (54.8) | 230 (72.6) | <0.001 |
| Antiplatelets | 49 (15.8) | 142 (44.8) | <0.001 |
| Aortic valve replacement | 198 (61.3) | 144 (45.4) | <0.001 |
| Aortic stenosis severity |  |  |  |
| Moderate | 107 (33.1) | 93 (29.3) | 0.343 |
| Severe | 216 (66.9) | 224 (70.7) |  |

Data are shown as mean ± SD or n (%). Baseline characteristics of the 317 eligible patients from the RED-CARPET study, stratified by the optimal cutoff point of triglyceride glucose index. TyG = triglyceride-glucose; BMI = body mass index; SBP=systolic blood pressure; DBP = diastolic blood pressure; FPG = fasting plasma glucose; HDL-C = high-density lipoprotein cholesterol; LDL-C = low-density lipoprotein cholesterol; TG = triglyceride; CHD = coronary heart disease.

*Additional file 1: Table S4.* Association between the TyG index and all-cause mortality in the moderate to severe AS patients with further adjustment for use of insulin and oral hypoglycemic agents*.

|  |  |  | Model 1 | |  |  | Model 2 | |
| --- | --- | --- | --- | --- | --- | --- | --- | --- |
|  | Events/Total |  | HR (95%CI) | *p* value |  |  | HR (95%CI) | *p* value |
| Continuous (per unit) | 84/317 (26.5) |  | 1.654 (1.119, 2.431) | 0.012 |  |  | 1.689 (1.116, 2.559) | 0.013 |
| Lower TyG index | 37/168 (22.0) |  | Reference | - |  |  | Reference | - |
| Higher TyG index | 47 /149 (31.5) |  | 1.802 (1.136, 2.867) | 0.012 |  |  | 1.761 (1.103, 2.821) | 0.017 |

Abbreviations: TyG, triglyceride-glucose; HR, hazard ratio; CI, confidence interval.

Model 1 Adjusted by sex, age, body mass index, low-density lipoprotein cholesterol, systolic blood pressure, diastolic blood pressure, smoking status, drinking status, aortic stenosis severity, pulmonary arterial hypertension, LV ejection fraction, bicuspid aortic valve, aortic valve replacement, diabetes mellitus, CHD, antiplatelets, and statins use.

Model 2 Further adjusted by model 1+ use of insulin, use of oral hypoglycemic agents.

^*^Firth’s penalized maximum likelihood Cox regressions were used.
